# Partial information decomposition as a unified approach to the characterization and design of neural goal functions

**DOI:** 10.1186/1471-2202-16-S1-P199

**Published:** 2015-12-18

**Authors:** Michael Wibral, William A Phillips, Joseph T Lizier, Viola Priesemann

**Affiliations:** 1 MEG Unit, Brain Imaging Center, Goethe University, Frankfurt 60528, Germany; 2 Ernst Strüngmann Institute for Neuroscience, Frankfurt 60528, Germany; 3 School of Natural Sciences, University of Stirling, Stirling FK9 4LA,UK; 4 School of Civil Engineering, The University of Sydney, Sydney, NSW 2006, Australia; 5 Department of Nonlinear Dynamics, Max Planck Institute for Dynamics and Self-Organization, 37077 Göttingen, Germany; 6 Bernstein Center for Computational Neuroscience, 37077 Göttingen, Germany

## 

In many neural systems anatomical motifs are found repeatedly in different places. Despite this repetition these motifs often seem to serve a perplexing variety of functions. A prime example is the canonical microcircuit, which is repeated across multiple cortical areas, but supports a variety of functions from sensory processing and memory to executive functions and motor control. The multiplicity of functions served by a single anatomical motif suggests a common, but more abstract, information processing goal underlying all the different functions. Identifying this goal from neural recordings is a key challenge in understanding the general principles of neural information processing. The apparent diversity of functions makes it clear that this common goal cannot be described using function-specific language (e.g. "edge filters"), but calls for an abstract framework. Here, information theory is the obvious candidate. Notable past approaches using information theoretic descriptions of neural goal functions suggested to maximize the mutual information between input and output [1], maximize the coherent mutual information that all the inputs share about the output [2], or, very generally, to minimize the free energy [3]. To facilitate these efforts, and to better dissect the implications of existing neural goal functions, we suggest to build on a recent progress in information theory, termed partial information decomposition (PID). PID allows to measure which of a set of inputs contributes either uniquely, redundantly or synergistically to the output of a (neural) processing unit [4-7], and which fraction of the output's entropy remains unexplained by the input set. We show how these measures can be used to identify an information theoretic footprint of a neural goal function. Most importantly, these measures can quantify how much of the information is modified rather than merely relayed when passing through the neural processor [8]. This shifts the focus from information transmission to more complex processing and allows a much better understanding of the (theoretical?) capabilities of a neuron or neural circuit. Using this approach we show how to better understand existing neural goal functions using PID measures and provide an information theoretic framework for the design of novel goal functions for artificial neural networks.
